# TRPV4 is activated by mechanical stimulation to induce prostaglandins release in trabecular meshwork, lowering intraocular pressure

**DOI:** 10.1371/journal.pone.0258911

**Published:** 2021-10-21

**Authors:** Takatoshi Uchida, Shota Shimizu, Reiko Yamagishi, Suzumi M. Tokuoka, Yoshihiro Kita, Rei Sakata, Megumi Honjo, Makoto Aihara

**Affiliations:** 1 Department of Ophthalmology, Graduate School of Medicine, the University of Tokyo, Tokyo, Japan; 2 Senju Laboratory of Ocular Science, Senju Pharmaceutical Co., Ltd., Kobe, Japan; 3 Department of Lipidomics, Graduate School of Medicine, the University of Tokyo, Tokyo, Japan; 4 Life Science Core Facility, Graduate School of Medicine, the University of Tokyo, Tokyo, Japan; Indiana University School of Medicine, UNITED STATES

## Abstract

Trabecular meshwork constitutes the conventional outflow pathway and controls intraocular pressure by regulating aqueous outflow. Mechanical stimulation has been studied as one of the triggers to regulate aqueous outflow in trabecular meshwork, but it is not well understood. We investigated that how transient receptor potential cation channel subfamily V member 4 (TRPV4) functions in human trabecular meshwork cells (HTMC) and affects intraocular pressure (IOP). HTMC were treated with TRPV4 siRNA, followed by incubation for 24 hours. We confirmed the suppression of *TRPV4* mRNA expression and the reduction of Ca^2+^ influx by the TRPV4 agonist GSK1016790A in TRPV4 siRNA-treated HTMC. TRPV4 siRNA-treated HTMC exhibited a significant reduction in Ca^2+^ influx and production of arachidonic acid and prostaglandin (PG) E_2_ induced by mechanical stretch, and direct activation of TRPV4 by GSK1016790A increased production of arachidonic acid, PGE_2_, and PGD_2_ and inhibited gel contraction. Furthermore, TRPV4-deficient mice had higher IOP than wild-type mice, and GSK1016790A administration lowered IOP. These results suggest that TRPV4 mediates the cellular response induced by trabecular meshwork stretch, leading to IOP reduction through the production of prostaglandins and inhibition of cell contraction. Targeting TRPV4 may have therapeutic benefits that lead to lowering IOP in glaucoma patients.

## Introduction

Glaucoma is a disease that cause visual defect due to injury of optic nerve and is a leading cause of blindness in modern society [1]. Evidence-based treatment for glaucoma, including normal tension glaucoma, is defined as lowering intraocular pressure (IOP) [2, 3]. Currently, IOP-lowering drugs mainly have a mechanism that suppress the production of aqueous humor from ciliary body or reduces aqueous humor drainage resistance via the uveoscleral route. However, the major route for aqueous humor drainage is the conventional outflow pathway, which is consist of trabecular meshwork (TM) and Schlemm’s canal [4]. It is an important issue to gain an understanding of the control mechanism of the conventional outflow pathway because promotion of aqueous humor drainage leads to a decrease in IOP. TM is sensitive to mechanical stimuli such as stretch and strain, and regulates IOP by altering aqueous humor outflow in response to various pressures [5–8]. However, the molecular mechanisms in TM that respond to IOP and regulate aqueous humor outflow are poorly understood.

Several ion channels have been identified as mechanosensors in TM, one of which is the transient receptor potential cation channel subfamily V member 4 (TRPV4). TRPV4 is involved in various biological functions and diseases, and activated by a variety of chemical and physical stimuli such as swelling, temperature, mechanical stimulation, low pH, and lipid mediators [9]. The first report of TRPV4 expressed in TM was described in 2014 as a ciliary mechanosensory channel [10]. Systemic TRPV4 agonist GSK1016790A treatment significantly reduced IOP in wild-type mice and IOP in TRPV4^−/−^ mice was elevated compared to that in control TRPV4^+/+^ animals [10]. On the other hand, different group reported that gene knockout and intraocular injection of GSK1016790A or TRPV4 antagonist HC-06 have no effect on IOP [11] and TRPV4 antagonists reduced IOP in chronic hypertensive eyes, but not in naive eyes [12]. Patel et al. showed that instillation of GSK1016790A significantly reduced IOP in C57BL/6J mice [13]. Thus, there is no consensus about the effect of TRPV4 in TM on IOP.

Mechanical stimulation has been reported to rapidly increase cPLA2 activity leading to the synthesis and release of lipid mediators [14–16]. Among the lipid mediators, prostaglandins (PGs), Lysophosphatidic acid and Sphingosine-1-phosphate are involved in IOP regulation [17–21]. In particular, PGF_2α_ receptor (FP) agonists are used to reduce IOP in glaucoma patients [17, 18, 22]. PGE_2_ and some PGE_2_ receptor (EP) agonists have been shown to have strong potential for IOP reduction [17, 18, 22, 23]. We have previously reported that mechanical extension stimulation on HTMC increases the production of arachidonic acid and PGE_2_ [16]. Therefore, we investigated the relationship between these lipid mediators released by mechanical stretch and TRPV4, and conducted a comprehensive analysis of lipid mediators generated by TRPV4 activation in TM.

In this study, we investigated how TRPV4 functions in mechanosensory transduction in TM and regulating IOP via PGs.

## Material and methods

### Cell culture

Unless otherwise stated, chemicals used in these studies were obtained from FUJIFILM Wako Pure Chemical Corporation (Osaka, Japan). All experiments were approved by the University of Tokyo’s ethics committee (10984-(4)) and comply with the Declaration of Helsinki. As previously reported [16], primary human trabecular meshwork cells (HTMC) were harvested and cultured. Briefly, corneal scleral rims recovered by Rocky Mountain Lions Eye Bank from donors with written consent for use in research by next of kin and stored in Optisol were sectioned and trabecular meshwork strips were isolated and cultured in cell culture dishes coated with collagen gel (Cellmatrix Type I-A, Nitta Gelatin Inc., Osaka, Japan). When HTMC spread from TM tissue, it was treated with collagenase to disperse the cells and seeded on dishes coated with 1 μg / mL fibronectin (CORNING, Corning, NY, USA) until further use. For subsequent experiments, HTMCs were used in 3 to 6 passages and grown on Trabecular Meshwork Cell Medium (ScienCell Research Laboratories, Carlsbad, CA), which contains 2% fetal bovine serum (ScienCell), 1% Trabecular Meshwork cell growth supplement (ScienCell), and 1% penicillin/streptomycin solution (ScienCell). Primary HTMC were identified and characterized by dexamethasone-induced *MYOC* expression as described previously [16]. Following the manufacturer’s instructions, HTMC were transfected with siRNA that specifically knockdown *TRPV4* (SASI_Hs01_00013361, SASI_Hs02_00354974) and MISSION siRNA Universal Negative Control #1 siRNA (Merck, Darmstadt, Germany) with MISSION siRNA Transfection Reagent (Merck).

### Quantitative real-time polymerase chain reaction (qPCR) analysis

HTMC was lysed using ISOGEN (Nippon Gene, Tokyo, Japan) 24 h after transfection with siRNA, total RNA was extracted and then reverse transcribed into Complementary DNA (cDNA) using the ReverTra Ace qPCR RT Master Mix with gDNA remover (Toyobo, Osaka, Japan). qPCR was performed with the Thermal Cycler Dice Realtime System (Takara Bio Inc., Shiga, Japan) with TB Green Premix Ex Taq^™^ II (Tli RNaseH Plus) (Takara Bio). The gene expression level of TRPV4 was standardized with that of GAPDH. The primer sequences used in qPCR were as follows: human *TRPV4* (forward, 5’-TGCATGCGCCACCATTTTTG-3’ and reverse, 5’-TATTGAGCACCGGCAAATCC-3’); human *glyceraldehyde-3-phosphate dehydrogenase* (*GAPDH*, forward, 5’-AATTCCATGGCACCGTCAAG-3’ and reverse, 5’-ATCGCCCCACTTGATTTTGG-3’). The values for *TRPV4* gene were normalized to the level of *GAPDH*.

### Ca^2+^ imaging and mechanical extension stimulation

HTMC untreated or treated with siRNA for 24 hours were seeded on 1 μg/mL fibronectin-coated cover glass chambers (Iwaki, Shizuoka, Japan) or STB-CH-24 (stretch chamber, STREX Inc., Osaka, Japan). HTMC were washed with PBS and incubated with 5 μM fluorescent Ca^2+^ indicator Fluo-8 AM (AAT Bioquest, Inc., Sunnyvale, CA) and 0.1% BSA (Sigma) for 20 minutes. The TRPV4 agonist GSK1016790A (10 nM) was added into the cover glass chamber on the fluorescence microscope (Keyence, Osaka, Japan). STB-CH-24 was placed on an STB-150 attached to a fluorescence microscope and subjected to a 30% single uniaxial elongation stimulus (extend for 1 s, stop for 3 s, return in 1 s) as reported [16]. The fluorescence intensity of Fluo-8 in images taken with a fluorescence microscope was determined using ImageJ software (https://imagej.nih.gov/ij/download.html) as described previously [16]. Briefly, fluorescence intensity was measured in all cells on images and the change ratio (F_T_/F_0_, F_max_/F_0_) was calculated using peak and basal values. F_T_, F_max_, and F_0_ represent the fluorescence intensity at that time, the maximum fluorescence intensity, and the fluorescence intensity before stimulation, respectively.

### Lipid analysis

Lipid analysis was performed as previously described [24]. After GSK1016790A treatment or stretch stimulation, HTMC supernatant was collected and stored at -80°C. Each sample was mixed with methanol in a 1: 1 ratio and 10 μL of internal standard solution was added. Lipids were extracted from the mixture and the resulting samples were analyzed with a Nexera ultra-high performance liquid chromatograph connected to a triple quadrupole mass spectrometer LCMS-8060 (Shimazu, Kyoto, Japan). Chromatographic separation was performed using a Kinetex C8 column (2.6 μm, 2.1 × 150 mm, Phenomenex, Torrance, CA). The mass spectrometer was operated in selected reaction monitoring mode for simultaneous detection of target lipid mediators, and quantitation was performed by internal standard calibration method using chromatographic peak areas, as described previously [24].

### Gel contraction assay

Cell contraction was evaluated using the Collagen Gel Culturing Kit (Nitta Gelatin, Inc., Osaka, Japan). Collagen type I, 10× MEM, and reconstitution buffer (pH 7.3) were mixed in a 7:1:1 ratio while cooling, and it was mixed with a cell suspension adjusted to 1 × 10^7^ cells/mL in a ratio of 9: 1. The mixture at a volume of 500 μL was dispensed into each well of 24-well plates and incubated at 37°C for 60 minutes. The gels were released from the wells and four gels were transferred to each 6 cm dish containing DMSO or GSK1016790A (10, 100 nM) and 5 mL Trabecular Meshwork Cell Medium. After 0, 12, 24, 48, and 72 hours, the dishes containing gels were photographed with a gel imaging device and the area of each gel was quantified with ImageJ software.

### Animals

We used TRPV4^+/+^ and TRPV4^-/-^ mice on the C57BL/6 background [25, 26]. All experiments were performed using 9- to 12-week-old mice. Food and water were available ad libitum. Animals were maintained in ordinary animal cages under constant 12-hour light/dark cycles. All procedures were performed in accordance with the ARVO Statement for the Use of Animals in Ophthalmic and Vision Research and approved by the Institutional Animal Research Committee of the University of Tokyo (P017-005).

### Mouse IOP measurements

C57BL/6 Wild type mice were intraperitoneally administered with vehicle (0.5% DMSO) or 0.25 mg/kg GSK1016790A 1.5 hours before IOP measurement. The mice were anesthetized by intraperitoneal injection of a mixture of ketamine (100 mg/kg, KETALAR; DAIICHI SANKYO PROPHARMA CO., LTD., Tokyo, Japan) and xylazine (10 mg/kg, Selactar; Bayer Holding Ltd., Tokyo, Japan) immediately before the measurement. IOP was measured using the microneedle method as previously described [27]. A microneedle made of borosilicate glass was placed in the anterior chamber and IOP was measured with a pressure transducer connected to the microneedle and recorded by a data acquisition and analysis system.

### Statistical analysis

Data were plotted as individual data and expressed as mean ± standard error (SE). Statistical analysis was performed using a two-tailed Student’s *t*-test or Dunnett’s test. *p* < 0.05 was considered a statistically significant difference.

## Results

### Effect of TRPV4 siRNA treatment on HTMC

**Fig 1:**
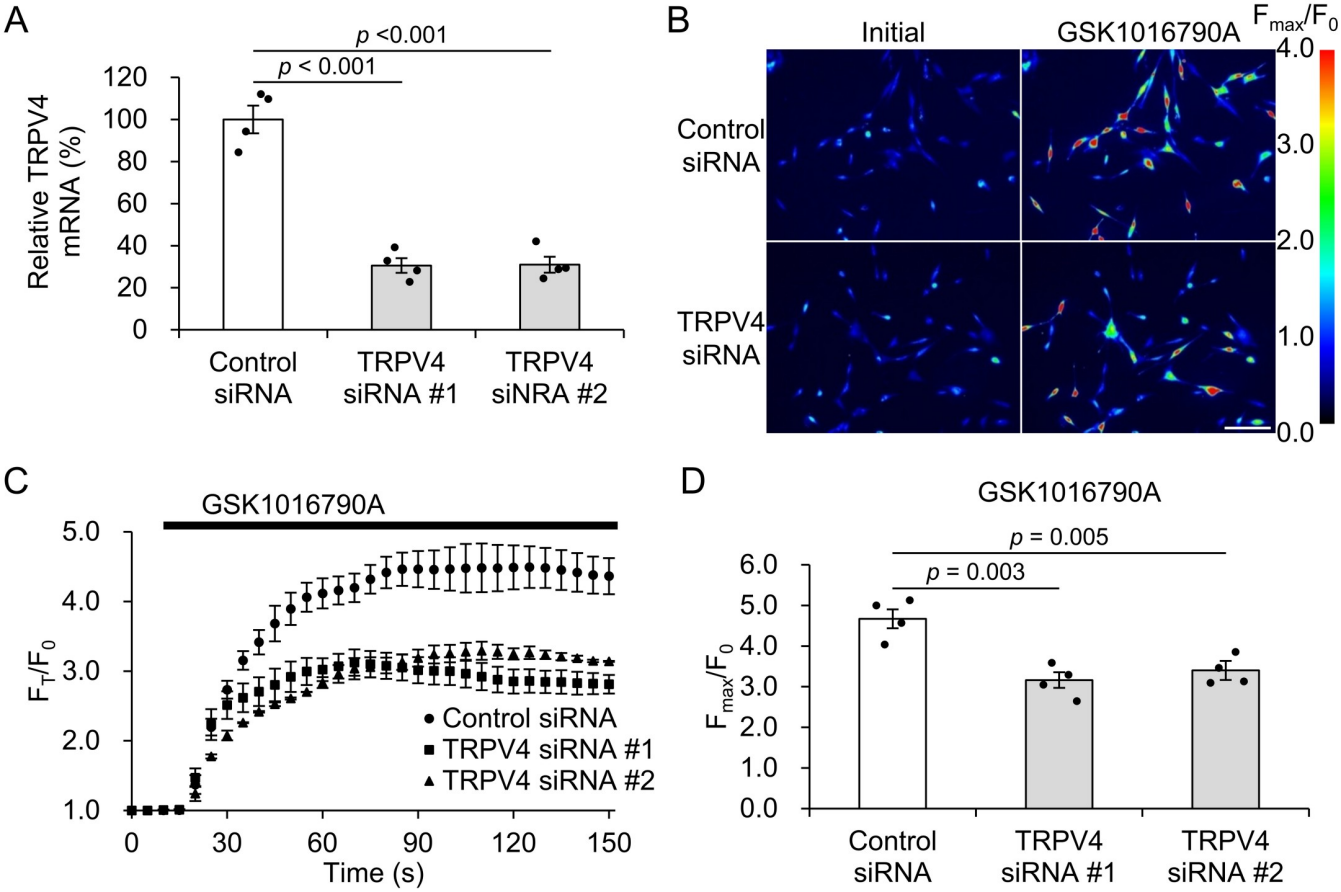
Knockdown of TRPV4 with siRNA in HTMC. (A) The knockdown of TRPV4 mRNA was confirmed by qPCR. Data are mean ± SE (n = 3). (B) Representative pseudo-color images of intracellular Ca^2+^ changes in control or TRPV4 siRNA-treated HTMC before and after stimulation with TRPV4 agonist GSK1016790A. Scale bar, 100 μm. (C, D) Quantification of GSK1016790A-induced intracellular Ca^2+^ elevation (change in Fluo-8 ratio) in control or TRPV4 siRNA-treated HTMC. Black bar represents GSK1016790A treatment. Data are expressed as the mean ± SE (n = 5).

To investigate the role of TRPV4 in the stretch-stimulated response of HTMC, we transfected primary HTMC with TRPV4 or control siRNAs. A significant decrease in TRPV4 mRNA levels was observed in TRPV4 siRNA-treated cells compared with that in control (siRNA#1, 30.6 ± 3.5%, and #2, 31.0 ± 3.8%, Fig 1A). In addition, we evaluated Ca^2+^ influx by the TRPV4 agonist GSK1016790A. In HTMC incubated with the Ca^2+^ indicator Fluo-8, TRPV4 siRNA treatment significantly suppressed the increase in intracellular Ca^2+^ by GSK1016790A (Control siRNA, 4.67±0.24 arbitrary units (A.U.), siRNA#1, 3.16 ± 0.19 A.U., and siRNA#2, 3.40 ± 0.17 A.U., Fig 1B–1D). These indicate that TRPV4 is expressed and functional in primary HTMC, and that siRNA transfection significantly suppresses TRPV4 at mRNA and functional levels. In subsequent experiments, we used TRPV4 siRNA#1, which has a higher KD efficiency.

### Mechanical extension stimulation induces Ca^2+^ influx through TRPV4 in HTMC

**Fig 2:**
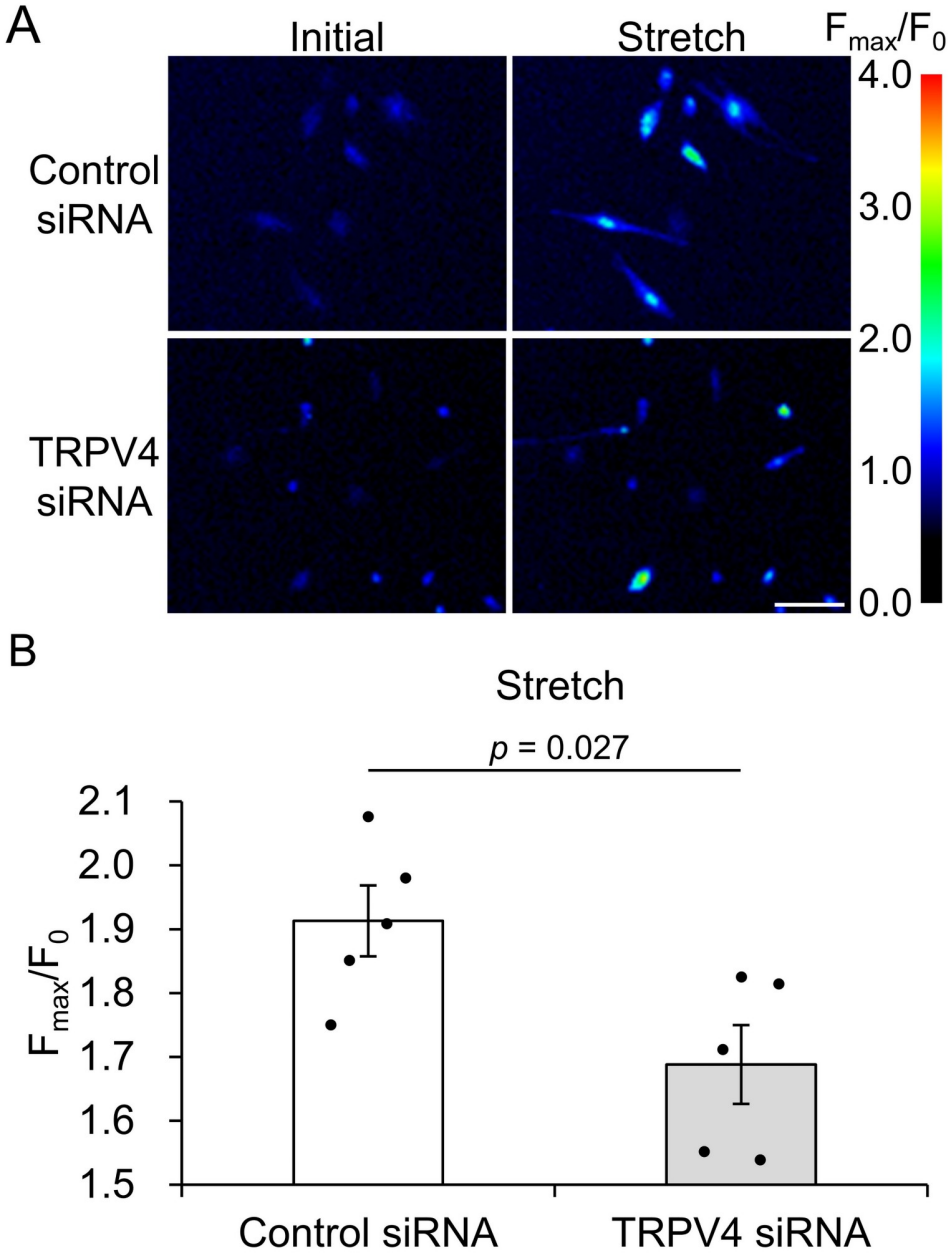
The effect of TRPV4 knockdown on intracellular Ca^2+^ changes induced by extension stimuli in HTMC. (A) Representative pseudo-color images of intracellular Ca^2+^ changes in control or TRPV4 siRNA-treated HTMC before and after mechanical extension stimuli. Scale bar, 100 μm. (B) Quantification of mechanical extension stimuli-induced intracellular Ca^2+^ elevation (change in Fluo-8 ratio) in control or TRPV4 siRNA-treated HTMC. Data are expressed as the mean ± SE (*n* = 5).

When stimulated mechanically, Ca^2+^ influx is induced in HTMC [12, 16]. To investigate whether TRPV4 is involved in Ca^2+^ influx due by mechanical extension stimulus, HTMC were seeded on silicon chambers coated with fibronectin and given a uniaxial extension. We found that the elevated intracellular calcium induced by mechanical extension was significantly inhibited in TRPV4 siRNA-treated cells compared to control siRNA-treated cells (Control siRNA, 1.91±0.06 A.U., and siRNA, 1.69±0.06 A.U., Fig 2A and 2B). This suggests that mechanical extension stimulus to primary HTMC caused an increase in intracellular Ca^2+^ through TRPV4 channels.

### TRPV4 mediates the release of arachidonic acid and PGE_2_ from HMC in response to mechanical extension stimuli

**Fig 3:**
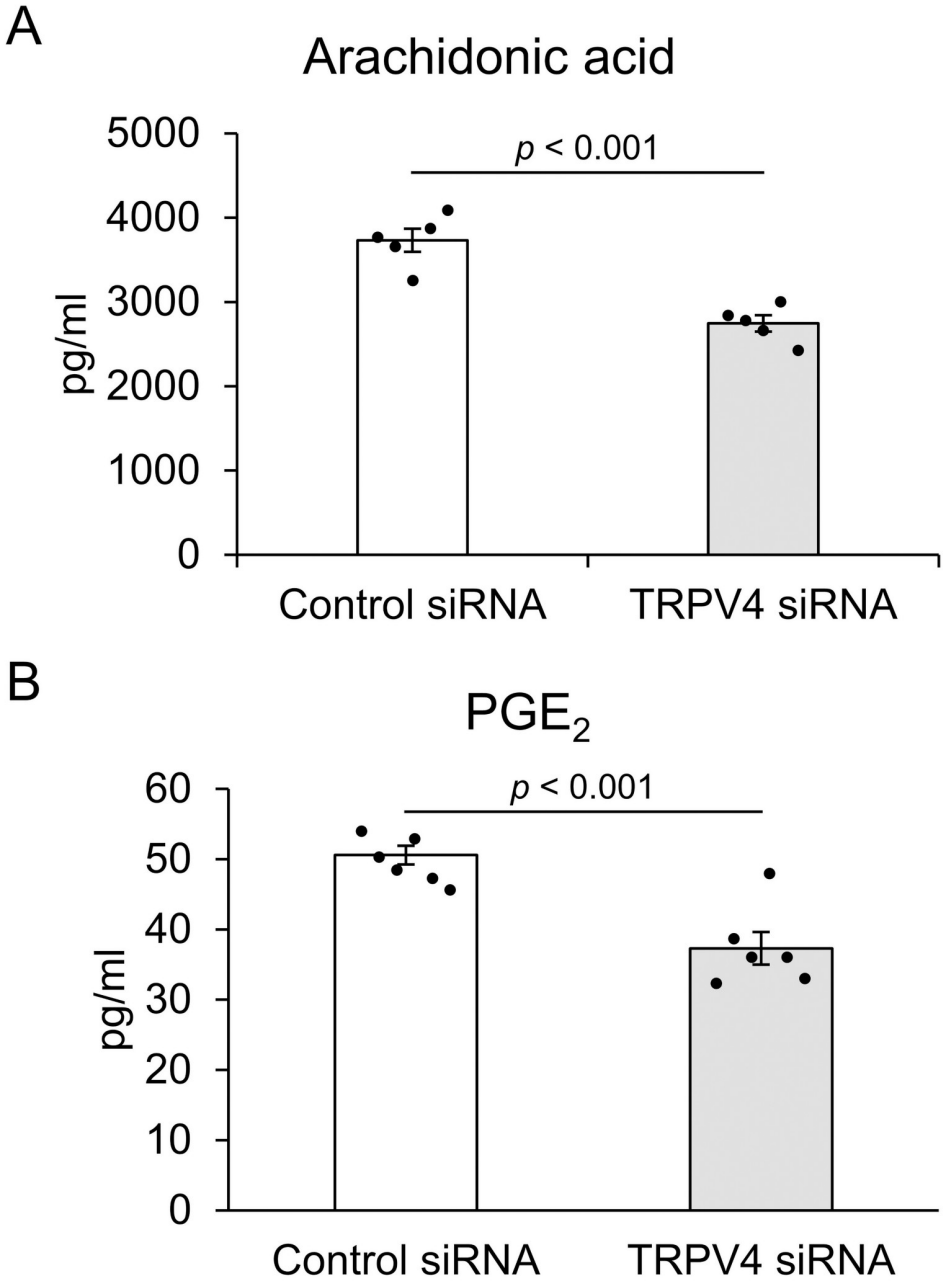
Effect of TRPV4 knockdown on enhanced arachidonic acid and PGE_2_ release by stretch in HTMC. The amount of arachidonic acid (A) and PGE_2_ (B) in cell supernatant 10 min after mechanical stretch stimulation in control or TRPV4 siRNA-treated HTMC. Data are expressed as mean ± SE (n = 3).

We have reported that the release of arachidonic acid and PGE_2_ from HTMC was enhanced by mechanical extension stimulation [16]. Therefore, in this study as well, we investigated the effect of TRPV4 on lipid mediators released by HTMC after mechanical extension stimulation by lipid analysis. Compared to control siRNA, TRPV4 siRNA treatment significantly reduced the release of arachidonic acid (Control siRNA, 3731.4±138.2 pg/ml, and siRNA, 2745.0±96.1 pg/ml, Fig 3A) and PGE_2_ (Control siRNA, 50.5±1.3 pg/ml, and siRNA, 37.3±2.3 pg/ml, Fig 3B) release by stretch stimulation in HTMC, indicating that TRPV4 mediated arachidonic acid and PGE_2_ release by extension stimulus in HTMC.

### GSK1016790A markedly enhanced the release of arachidonic acid, PGE_2_, and PGD_2_ from HTMC

**Fig 4:**
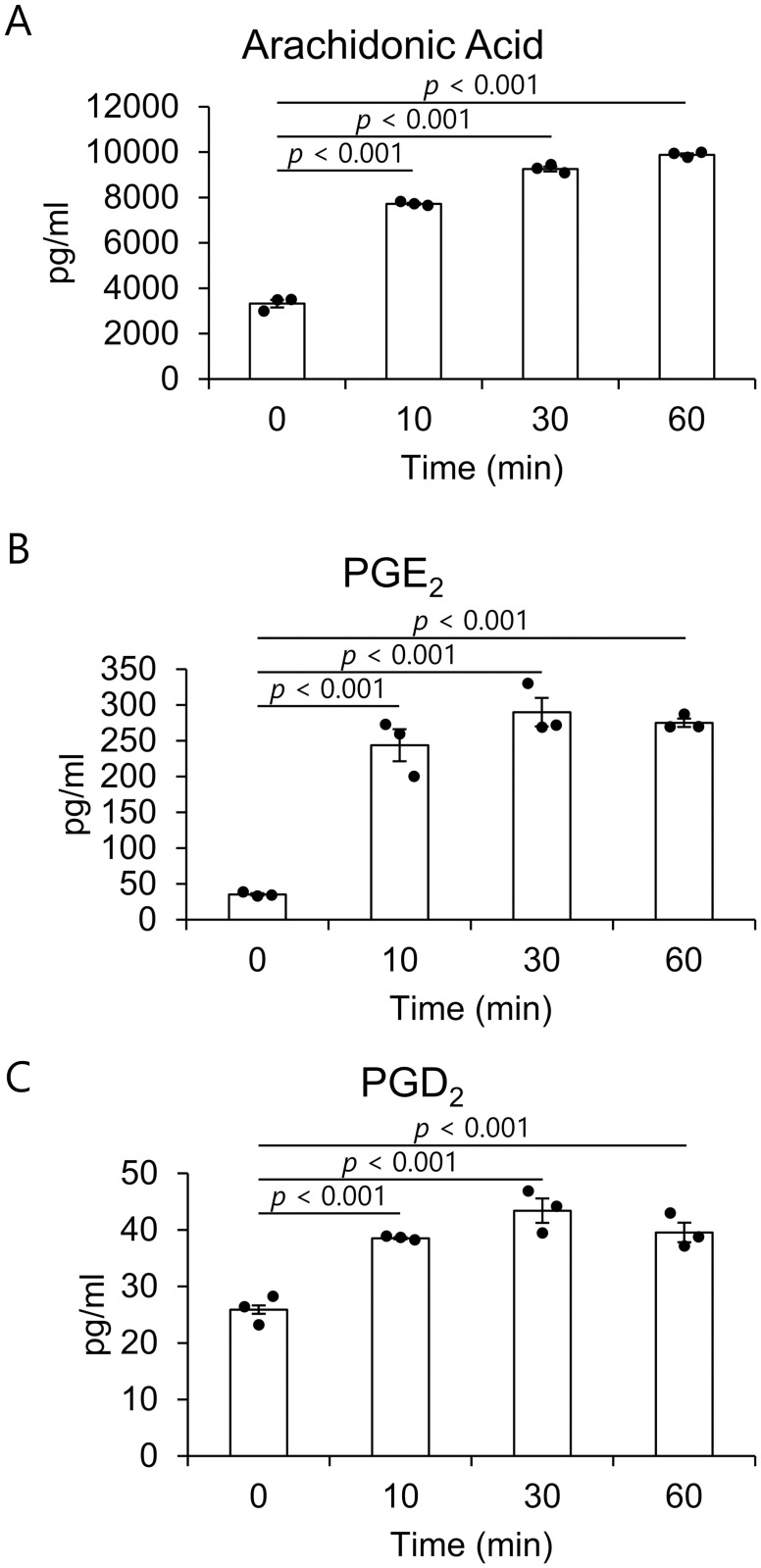
GSK1016790A promotes the release of PGD_2_ in addition to arachidonic acid and PGE_2_ from HTMC. The amount of arachidonic acid (A), PGE_2_ (B) and PGD_2_ (C) in the culture supernatant of HTMC at each time after GSK1016790A treatment. Data are expressed as the mean ± SE (n = 3).

To examine the effect of direct TRPV4 activation on the release of lipid mediators, we investigated lipid mediator levels in HTMC supernatant after TRPV4 agonist GSK1016790A exposure. Activation of TRPV4 by GSK1016790A significantly increased the release of PGD_2_ from HTMC, in addition to arachidonic acid and PGE_2_ (Fig 4A–4C). The amount of arachidonic acid at 0, 10, 30, and 60 minutes after GSK1016790A treatment ware 3322.6±166.2, 7716.5±48.2, 9256.0±104.5, and 9881.6±66.5 pg/ml, and those of PGE_2_ were 35.2±1.7, 243.8±22.3, 289.9±19.9, and 275.1±5.8 pg/ml, and those of PGD_2_ were 25.9±0.7, 38.5±0.2, 43.4±2.2, and 39.5±1.7 pg/ml, respectively. Lipid mediators, including PGF_2α_ or EET, were not detected. These results suggest that arachidonic acid metabolites PGE_2_ and PGD_2_ were induced by TRPV4 activation.

### Effects of GSK1016790A on collagen gel contraction

**Fig 5:**
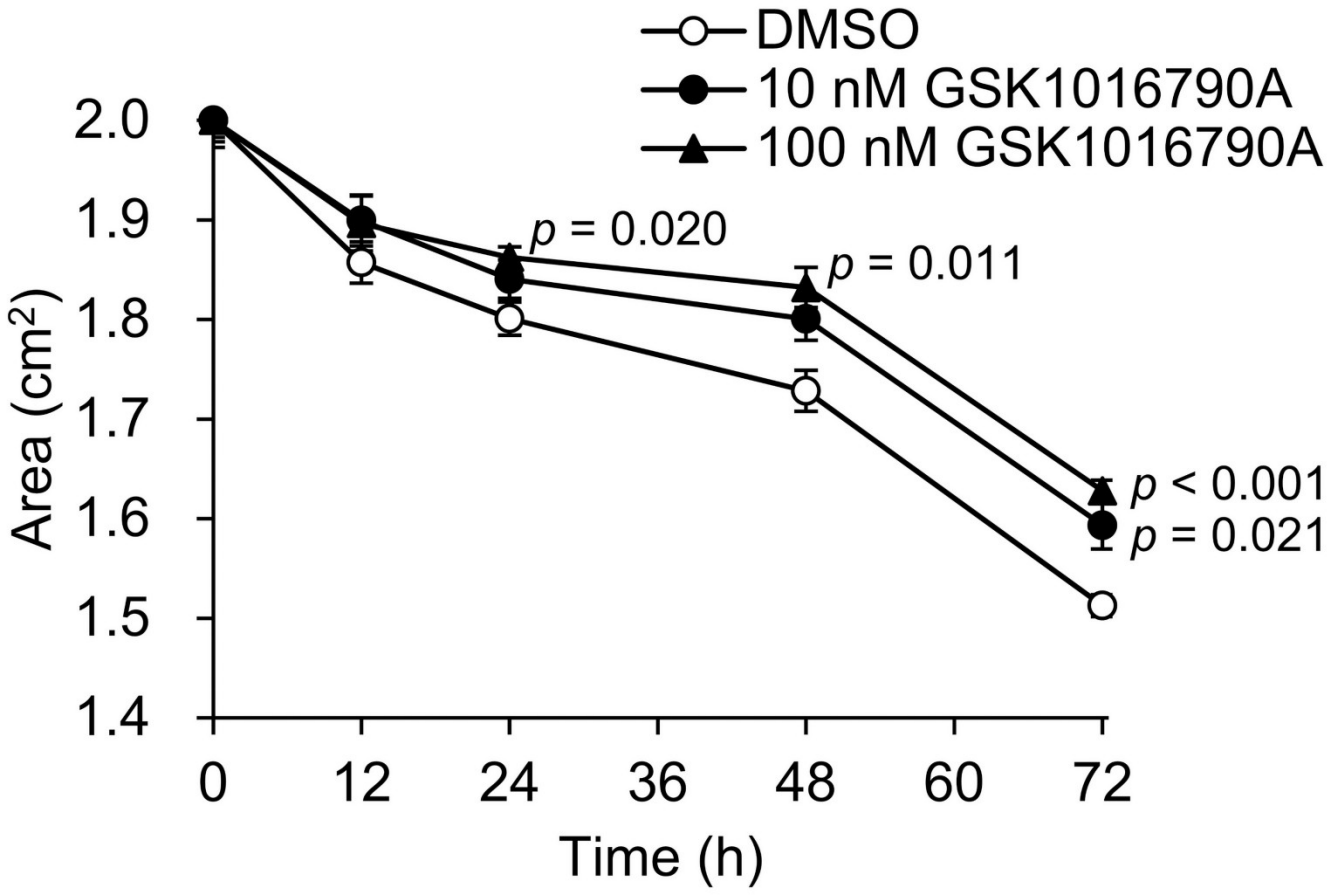
Effects of GSK1016790A on contraction of HTMC. Change rate in area of the collagen gels in presence of DMSO, 10 or 100 nM GSK1016790A were measured. Data are expressed as the mean ± SE (n = 4).

To evaluate the effect of TRPV4 activation on HTMC contraction, we conducted an *in vitro* collagen gel contraction assay. Compared with controls, 10 nM GSK1016790A at 72 hours and 100 nM GSK1016790A at 24, 48, 72 hours significantly inhibited collagen gel contraction (Fig 5), suggesting that TRPV4 activation inhibited the contractility of HTMC.

### TRPV4 regulate IOP in mouse

**Fig 6:**
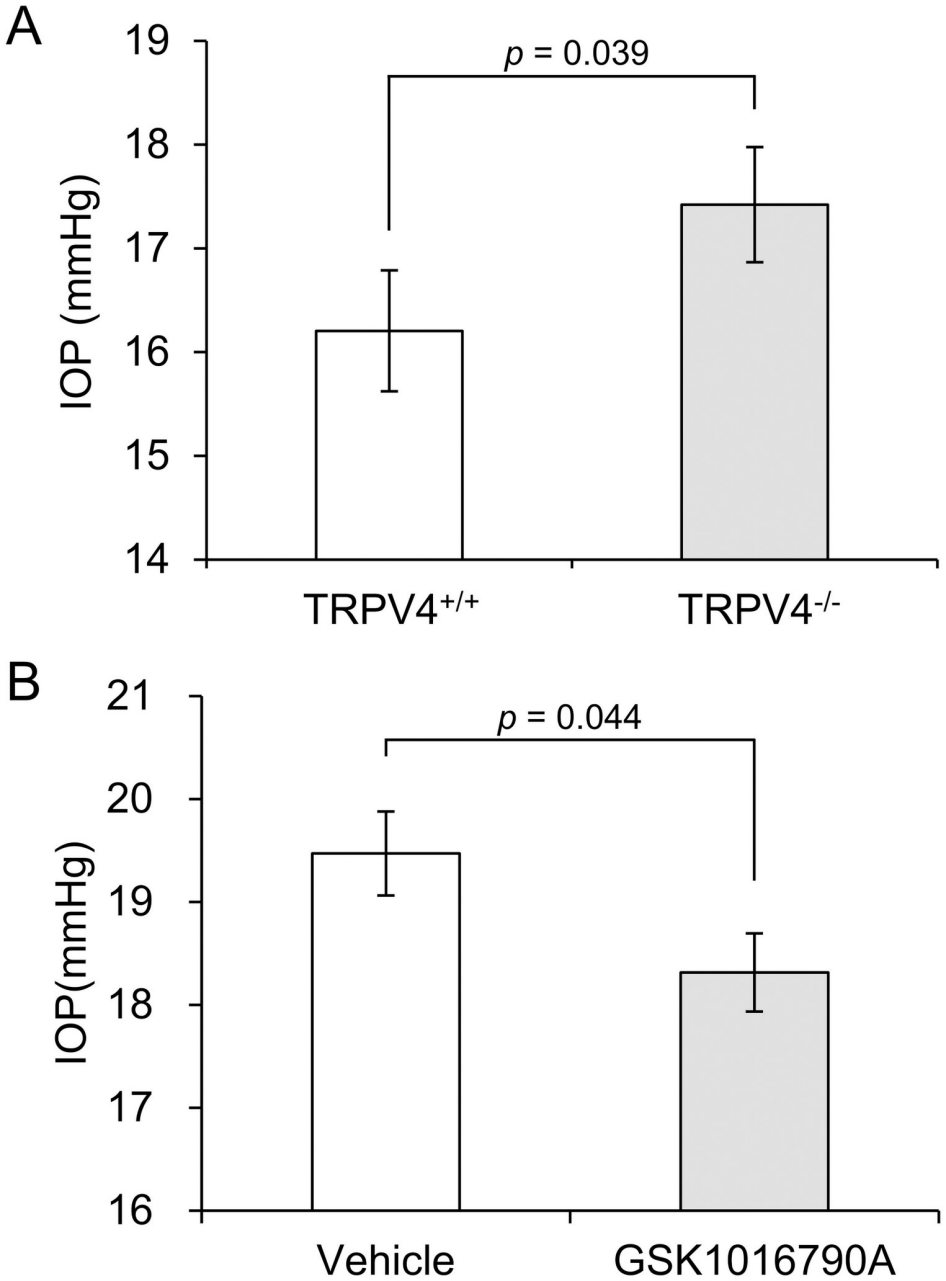
TRPV4 regulates intraocular pressure in mice. (A) Intraocular pressure (IOP) in nine to twelve-week-old TRPV4^+/+^ and TRPV4^−/−^ mice. IOP was measured using the microneedle method. Data are expressed as the mean ± SE (n = 22–24). (B) TRPV4 agonist GSK1016790A lowers IOP in C57BL/6 Wild type mice. Mice were treated with vehicle (0.5% DMSO) or 0.25 mg/kg GSK1016790A. IOP was measured using the microneedle method 1.5 h after intraperitoneal injection. Data are expressed as the mean ± SE (n = 22).

Given that TRPV4 responds to mechanical stretch stimuli and its activation inhibits cell contraction in TM cells, loss or activation of TRPV4 may affect IOP. Comparing IOP of *TRPV4*^+/+^ and *TRPV4*^-/-^ mice using microneedle method, *TRPV4*^-/-^ mice exhibited elevated IOP compared with WT *TRPV4*^+/+^ mice (17.4 ± 0.6 vs 16.2 ± 0.6 mmHg, Fig 6A). To examine the effect of TRPV4 activation on IOP, we measured IOP after administration of GSK1016790A to wild-type C57BL/6 mice. IOP was significantly decreased by systemic GSK1016790A administration (19.5 ± 0.4 vs 18.3 ± 0.4 mmHg, Fig 6B).

## Discussion

In this study, we firstly revealed that TRPV4 was involved in mechanical stretch-induced Ca^2+^ influx and PGE_2_ release from HTMC, and that TRPV4 agonist GSK1016790A promoted PGE_2_ and PGD_2_ release, and suppressed cell contraction in HTMC. *In vivo*, TRPV4^-/-^ mice had higher IOP than wild-type mice, and GSK1016790A administration in normal mice significantly decreased IOP compared with vehicle. These results collectively indicate that TRPV4 plays an important role in cellular response to mechanical stretch, regulating TM contraction and IOP. Therefore, TRPV4 has potential as a therapeutic target for IOP control and glaucoma.

We have previously reported that uniaxial stretch stimulation caused enhanced release of arachidonic acid and PGE_2_ from HTMC [16]. This study showed that TRPV4 was involved in the release of lipid mediators induced by stretch stimulation (Fig 3), and direct TRPV4 activation with GSK1016790A caused an increase in PGD_2_ release as well as arachidonic acid and PGE_2_ (Fig 4) in HTMC. In Ca^2+^ imaging results, GSK1016790A elicited a greater change in fluorescence intensity than stretch stimulation. This strong Ca^2+^ influx may release PGD_2_ in addition to PGE_2_ due to the large release of arachidonic acid, the most important source of PGs. This prostaglandin release triggered by the increase of intracellar Ca^2+^ is also found in other cells, including chondrocytes [28], macrophages [29], and vascular endothelium [30]. Previously, it has been reported that PGE_2_ and PGD_2_ reduced IOP, respectively [17, 18, 22, 23, 31–34], and recently EP2-targeted new glaucoma drug has been launched in Japan. Also, the combination of PG subtype activation, i.e. EP3 and FP dual agonist, may be a potent anti-glaucoma strategy [35, 36]. These reports suggest that the IOP reduction by TRPV4 may be caused by the release of PGE_2_ and PGD_2_.

TRPV4 is activated not only by extension and temperature and pharmacological agonists GSK1016790A, but also by endogenous agonists. Epoxyeicosatrienoic acid (EET) is a metabolite of arachidonic acid and functions as an endogenous final activator of TRPV4 [37]. In the lipid analysis of this study, EETs were also included in the analysis target, but not detected. This results suggest that stretch-generated arachidonic acid tends to be metabolized to PGs rather than EETs in TM cells.

Piezo1 channel, as well as TRPV4, mediated intracellular response of mechanical expansion in HTMC [16]. In urothelial cells, the functional role of Piezo1 and TRPV4 were compared, suggesting that Piezo1 senses stretch stimuli over a wider range than TRPV4, and that Piezo1 is more sensitive to stretch stimuli than TRPV4 [38]. Due to the difference in siRNA knockdown efficiency and agonist or antagonist specificity, it is not possible to directly compare the functions of TRPV4 and Piezo1 in HTMC, and it is not clear whether there are differences in sensitivity to stretch stimuli or which is more important. Further research is needed to determine the difference between TRPV4 and Piezo1 in HTMC, such as using knockout cells or double knockout cells for each channel.

There are several reports about the effect of TRPV4 on IOP. Luo et al. confirmed IOP was significantly reduced by systemic GSK1016790A treatment in wild-type C57BL/6 mice, and showed that TRPV4^-/-^ mice had elevated IOP compared to TRPV4^+/+^ mice [10]. We also confirmed that activation of TRPV4 reduced IOP in mice, and the IOP of TRPV4^-/-^ mice was higher than that of TRPV4^+/+^ mice (Fig 6). However, Jo et al. showed that intravitreal injections of agonists did not affect IOP levels in TRPV4^+/+^ or TRPV4^-/-^ tissues, and in addition, the IOP of TRPV4^+/+^ mice was not significantly different from that of TRPV4^-/-^ mice [11]. Patel et al. demonstrated that topical GSK1016790A treatment significantly lowered IOP in C57BL / 6J mice [13]. To measure the IOP, we used the microneedle method, while they used Tonolab. The microneedle method, which measures by inserting the needle directly into the anterior chamber, is more accurate than Tonolab. Not only the measurement method, but also the sample size, administration method, and animal age were different. Aging has been shown to be associated with increased TM stiffness [39], thus affecting the regulatory function of TRPV4.

Our findings showed an important role for TRPV4 in controlling IOP after TM stretch, but it may not be possible to extrapolate the results obtained in the mouse eye directly to the human eye. This is because the distribution and expression patterns of receptor subtypes may differ between mice and humans in ocular tissue. Previous reports have shown that the pattern of expression of EP receptor subtypes in TM is similar in humans and mice [40]. Therefore, the reduction of IOP in mice by commercially available PG analog is similar to that in humans [41]. Although expression levels of TRPV4 have not been compared, its expression has been confirmed in mouse and human eye TMs [12], so TRPV4 agonist may act in human TM. This study focused on lipid mediators and the resulting cellular contraction in TM cells. Cytoskeletal alteration and fibrotic responses in TM are also considered to be responsible for the pathology of glaucoma [42, 43]. Patel et al. showed that TRPV4 in TM plays an important role in regulating IOP by activating endothelial NOS (eNOS or NOS3), which is one of the nitric oxide synthases (NOS), and producing nitric oxide (NO) [13]. TRPV4 is expressed in various different ocular tissues. Therefore, it is important to consider comprehensively whether calcium influx induced by activation of TRPV4 causes cell contraction, cytoskeletal changes, or fibrotic responses via release of arachidonic acid and prostaglandins, production of NO by activation of eNOS, or other signaling in ocular tissues including TM.

In conclusion, we found that mechanical stretch stimulation induced Ca^2+^ influx followed by PGE_2_ release via TRPV4 in HTMC, and that this mechanical stretch stimuli-induced cellular response was also elicited by TRPV4 activation, inhibiting cell contraction and regulating IOP. Based on the results of our studies, TRPV4 is an important regulator of trabecular meshwork, suggesting that pharmacological activation of TRPV4 may lead to IOP reduction. TRPV4 activation in trabecular meshwork by agonists such as GSK1016790A could be a novel treatment for glaucoma.
